# The application of the surface energy based solubility parameter theory for the rational design of polymer-functionalized MWCNTs†
†Electronic supplementary information (ESI) available. See DOI: 10.1039/c8cp07411a


**DOI:** 10.1039/c8cp07411a

**Published:** 2019-02-14

**Authors:** Pablo Quijano Velasco, Kyriakos Porfyrakis, Nicole Grobert

**Affiliations:** a Department of Materials , University of Oxford , Parks Road , Oxford , OX1 3PH , UK . Email: pablo.quijanovelasco@materials.ox.ac.uk ; Email: nicole.grobert@materials.ox.ac.uk ; Email: kyriakos.porfyrakis@materials.ox.ac.uk ; Tel: +44 (0)1865 283720 ; Tel: +44 (0)1865 273724; b Williams Advanced Engineering , Grove , Oxfordshire , OX12 0DQ , UK

## Abstract

The surface energy based solubility parameters theory was applied to model the degree of polystyrene-functionalisation of MWCNTs in six different organic solvents. The experimental characterization of the polymer-functionalized MWCNTs is consistent with the predictions of this model providing a breakthrough towards the rational design of functionalized MWCNTs based on thermodynamic parameters.

## Abstract 2

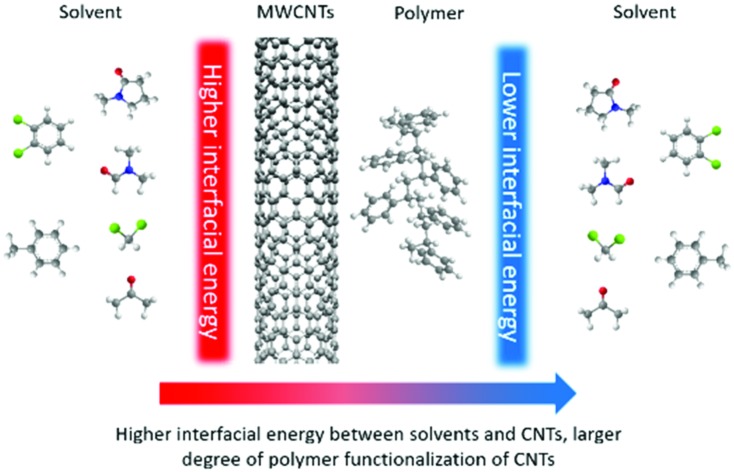
Solubility parameter theories can be used to model the degree of polymer functionalization of MWCNTs in different solvent media.

## 

Carbon nanotubes (CNTs) are found at the boundary between the molecular and the macroscopic scale granting them the ability to interact strongly with molecules through non-covalent interactions.^1^,^2^ Since their discovery, the non-covalent chemical functionalization of CNTs with polymers has been widely exploited to create dispersions in aqueous^3^,^4^ and organic media,^4^,^5^ to improve the surface interactions between CNTs and composite matrices,^6^,^7^ and for the synthesis of self-assembled hierarchical nanostructures.^8^ Nonetheless, most of the reports in the literature are based on the success of the functionalization for a particular application and there is not a general attempt to provide conceptual functionalization models that take into account the underlying interactions between the organic molecules and the CNTs. In order to unlock the full potential of the rational design of materials based on functionalized CNTs it is key to provide synthetic guidelines based on the current understanding of the molecular interactions that occur at the interface of the CNT and the organic molecule.

Two decades ago, O'Connell *et al.*^9^ proposed what today is the accepted mechanism for the non-covalent polymer functionalization of CNTs. In their study, they argued that the driving force for the functionalization of single-wall carbon nanotubes (SWCNTs) with polystyrene sulfonate and polyvinyl pyrrolidone in water was the unfavourable interfacial energy between the solvent and the CNTs. The authors explained that these unfavourable interactions between SWCNTs and water were reduced due to the surface functionalization of the SWCNTs with the water-soluble polymers. This same argument was used by Meuer *et al*.^4^ to provide an explanation for the larger degree of polymer functionalization of multi-wall carbon nanotubes (MWCNTs) with poly(diethylene glycol) monomethyl ether methacrylate observed when water was used as the solvent medium in comparison to tetrahydrofuran.

An implication of this hypothesis is that the degree of polymer functionalization of CNTs is dependent on the adhesive interactions between the solvent and the surface of the CNTs. Therefore, if we estimate these interactions we could potentially predict the degree of polymer-functionalization of CNTs in different media. The Hildebrand and Hansen solubility parameter (HSP) theories are good candidates to be used to model the interactions between CNTs and solvents, due to their success to estimate the solubility of a wide variety of solutes in organic solvents by calculating the difference of their cohesive energy densities. In fact, these theories have been widely used in the paint industry to design successful mixtures of binders, pigments and solvents,^10^ which represent conceptually an analogous problem as the polymer functionalization of CNTs.

The Hildebrand and Hansen solubility theories have already been applied with certain amount of success to find solvents for the efficient dispersion of different CNTs in organic media.^11^–^15^ In particular, the studies made by the Coleman research group have suggested that calculating the solubility parameters based on surface energy values provides a more accurate representation of the interactions between CNTs and solvents.^11^,^16^ Recently, these theories have been also used to evaluate the interactions of different surface functionalized SWCNTs with different polymer matrices^17^ and to find suitable polymers for the production of polymer-stabilized 2D-materials by the exfoliation of inorganic layered compounds such as graphite, boron nitride and molybdenum disulfide.^18^ To the best of our knowledge there are no reports on the application of these theories to evaluate the effect of solvents on the degree of polymer functionalization of CNTs.

Herein we report application of the solubility parameter theories based on surface energies (as derived by the Coleman research group)^11^,^16^ to estimate the degree of polystyrene-functionalization of MWCNTs in six different solvents. To demonstrate this, we compared the polymer weight loss observed in the thermogravimetric analysis (TGA) of the functionalized MWCNTs and the difference between the surface energy based solubility parameters (SEBSP) of the pristine MWCNTs and each of the solvents. For this study we used the solubility parameter values for MWCNTs reported by Lim *et al.*^14^ that were determined through an inverse gas chromatography procedure. The full details for the calculation of the SEBSP of MWCNTs and solvents can be found in the ESI† (Table S1).

A typical procedure for the non-covalent polymer-functionalization of MWCNTs with polystyrene consisted in ultrasonicating for 1 hour a mixture of 20 mg of MWCNTs, 200 mg of polystyrene (*M*_n_ = ∼3333 g mol^–1^ full characterization details available in the ESI†) in 20 ml of solvent. Afterwards the mixture was vacuum filtered and washed 3 times with 10 ml of solvent. To ensure that all the free polymer present in the sample was removed, the solid was re-dispersed in 20 ml of solvent and ultrasonicated for 15 minutes, filtered and washed 3 times with 10 ml of solvent. This procedure was repeated three times. The liquid phase obtained after each of the redispersion steps was evaporated under vacuum and the residue was used for NMR analysis.

Fig. 1 features the NMR spectrum from the filtrates after each step of the functionalization procedure from a sample where acetone was used as the solvent medium. It can be observed that the signal corresponding to polystyrene protons decreases subsequently after each filtration until it disappears in the last step. This behaviour is observed regardless the solvent used during the functionalization (Fig. S3, ESI†). Considering that polystyrene is soluble in all solvents used in this study we can be certain that we are removing the free polystyrene from the sample and the remaining polymer is bound to the surface of the MWCNTs by non-covalent interactions.

**Fig. 1:**
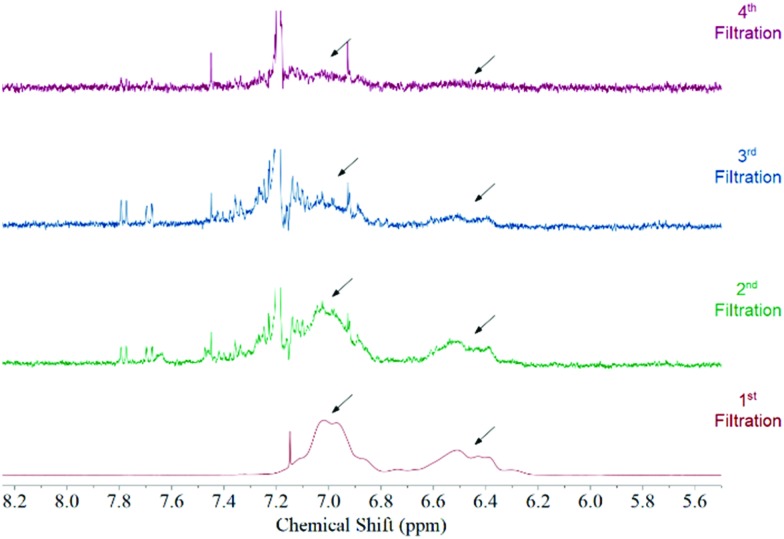
NMR spectrum of the filtrates after each washing step of the functionalization procedure. This spectrum was taken from a sample where acetone was used as the solvent. The arrows point to the position of polystyrene aromatic protons. No polymer can be observed after the fourth redispersion.

For TGA measurements, the solid samples were preheated to 90 °C under vacuum for 12 hours to eliminate any solvent residues. The experiments were run under nitrogen atmosphere to ensure the selective decomposition of the polymer. In this way we could quantitively assess the degree of polymer functionalization in each sample. The values of polymer weight loss were taken at 650 °C, after the polymer had decomposed completely. A typical TGA curve of the MWCNTs, polystyrene and the polystyrene functionalized MWCNTs is presented in Fig. S4, ESI.†

The experimental results are summarized in Table 1. One can observe that there is a dependence between the degree of non-covalent polymer functionalization and the solvent used during the experiments. The impact that the solvent has on the degree of polymer-functionalization is significant, to the extent that a three-fold increase in the polymer weight loss was observed when the solvent medium was changed from *o*-DCB to acetone. Furthermore, the three solvents in which the MWCNTs presented the lowest degree of polymer functionalization (DMF, NMP, *o*-DCB) have been widely regarded as good solvents for the dispersion of CNTs.^19^,^20^ The fact that these solvents presented the lowest degree of polymer functionalization is consistent with O’Connell *et al.* hypothesis, since the thermodynamic driving force for the polymer functionalization would be reduced due to the favourable interactions between the MWCNTs and these solvents. Transmission electron microscopy (TEM) was used to provide further evidence for the polymer functionalization of the MWCNTs, but no conclusive evidence was found due to the presence of other amorphous carbon materials in the pristine MWCNTs (Fig. S5, ESI†).

**Table 1:** Summary of the polymer weight loss for each solvent from TGA characterization, solvent-MWCNT Hildebrand SEBSP difference and solvent-MWCNT Hansen SEBSP *R*_a_ distance

Solvent	Mean weight loss [%]	Solvent-MWCNT Hildebrand SEBSP difference (mJ^1/2^ m^–1^)	Solvent-MWCNT Hansen SEBSP *R*_a_ distance (mJ^1/2^ m^–1^)
*o*-DCB	5.3 ± 1.0	6.9	2.6
NMP	8.6 ± 2.9	5.5	3.6
DMF	10.0 ± 0.2	6.8	4.6
DCM	11.1 ± 1.4	10.0	3.4
Toluene	11.7 ± 1.2	9.4	3.5
Acetone	15.9 ± 0.5	12	4.4

We can also note from these results that the difference in the degree of functionalization cannot be attributed to the interactions between the solvent and the polymer. If this was the case, solvents that present favourable interactions with polystyrene (*i.e.* good solvents) such as *o*-DCB, toluene and DCM^21^ would present a lower degree of functionalization in comparison to poor solvents such as DMF^22^ and acetone.^21^ This condition is only observed for *o*-DCB, and in fact one of the three solvents with the lowest degree of functionalization is considered a poor solvent for polystyrene. These observations suggest that the interactions between the MWCNTs and the solvents is the major driving force for the polymer functionalization.

As a first approach to understand the impact that the solvent medium has on the degree of polymer-functionalization of MWCNTs, we compared the difference of surface energies between each solvent and MWCNTs with the polymer weight loss observed in the TGA experiments. Previously Hughes *et al.*^16^ derived the following equation based on Hildebrand solubility parameter theory to predict the enthalpy of mixing between 1-D materials and solvents, based on the difference of their surface energies:1
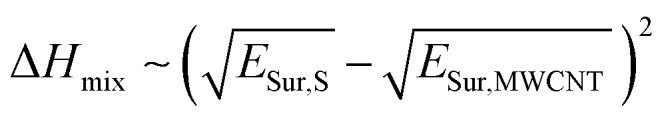



According to this equation, the solvents that present similar surface energies to MWCNTs would present more favourable adhesive interactions with their surface. In this way, we would expect that the degree of polymer-functionalization should decrease when the surface energy mismatch between the solvent and the MWCNTs is small.

It can be observed in Fig. 2a that there is a rough trend for solvents with smaller surface energy difference to present a lower degree of functionalization. However, this surface energy analysis does not fully explain the change in the degree of polymer functionalization of MWCNTs in different solvents. For example, according to this theory the degree of functionalization in *o*-DCB should be lower in comparison to the one observed in NMP. However, the opposite trend is observed experimentally.

**Fig. 2:**
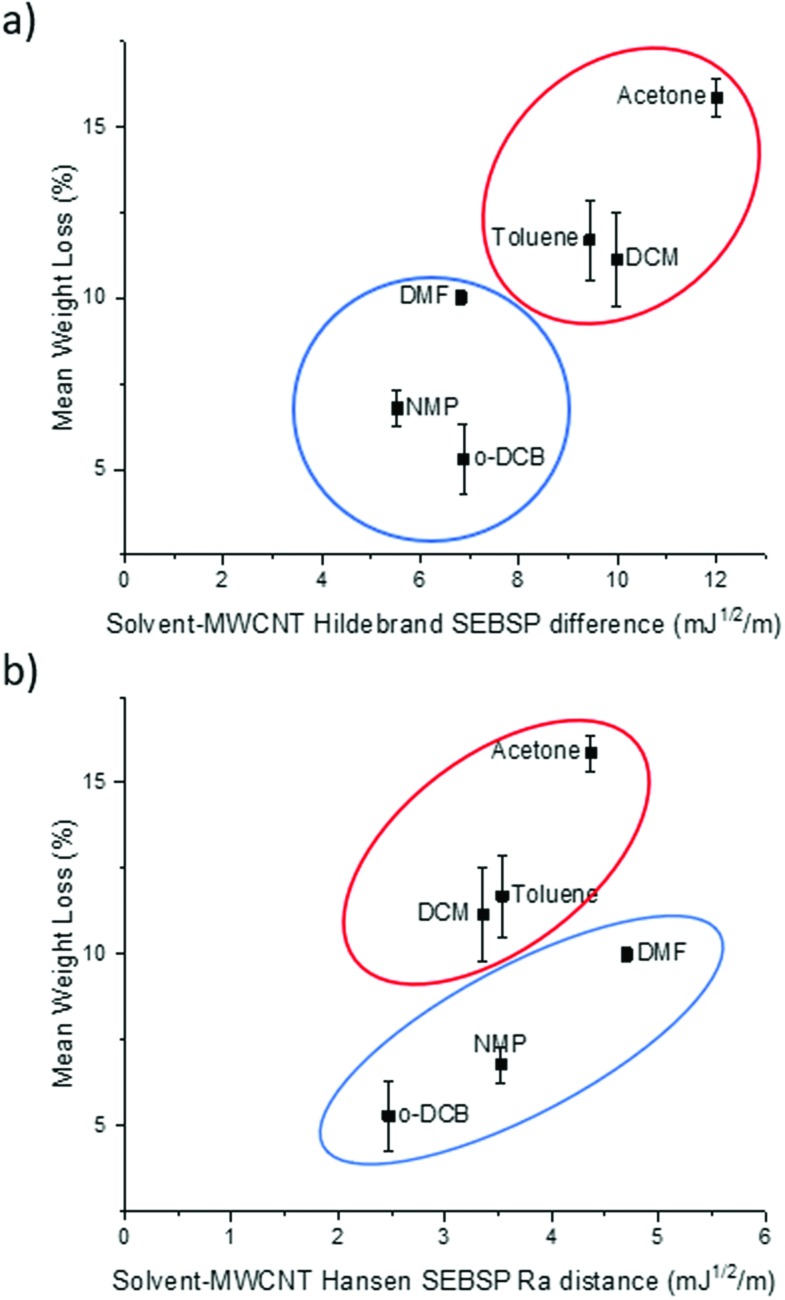
Plot of the (a) Hildebrand SEBSP difference and (b) Hansen *R*_a_ distance between the solvents and MWCNTs, against the mean polymer weight loss measured by TGA.

Using the surface energies of the solvents as a parameter to predict the degree of polymer functionalization of MWCNTs is an oversimplification since the amount of polymer wrapping will be determined by the intermolecular interactions that are present in the interface of the solvent molecules and the nanomaterial. A more accurate representation of this interactions may be obtained by applying the surface energy based solubility parameter theory derived by Bergin *et al*.^11^ This theory is analogous to the HSP, and it is based on the premise that the surface energy (*E*_S,Total_) of a molecule arises from the sum of three different intermolecular interactions. These three contributions are the dispersive interactions related to van der Waals forces (*E*_S,D_), polar interactions related to dipole–dipole electrostatic forces (*E*_S,P_) and hydrogen bonding interactions related to electron exchanges between a donor–acceptor pairs (*E*_S,H_).^11^,^23^ Thus, the total surface energy of a substance is defined as:

2 *E*_S,Total_ = *E*_S,D_ + *E*_S,P_ + *E*_S,H_

Each of the energy contributions have a related solubility parameter that is defined as:3
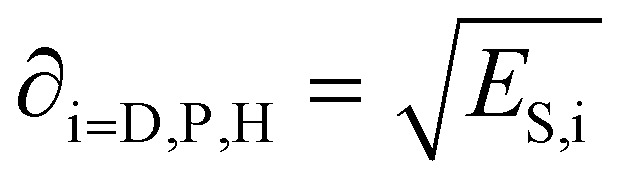



If these solubility parameters are plotted in a 3-dimensional space, the distance between each point will correlate with the difference between their surface energies. Bergin *et al.*^11^ propose to calculate this distance parameter (*R*_a_) between a solvent and a CNT as follows:4
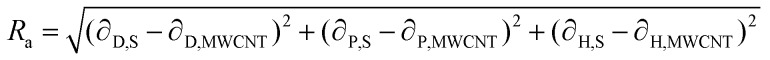



Thus, the enthalpic contribution to the free energy is minimized when the surface energy solubility parameters of the solute and solvent are similar to each other.^11^

To obtain a more accurate prediction of the degree of polymer functionalization we calculated the *R*_a_ distance of each solvent to the HSP value of MWCNTs using eqn (4). In Fig. 2b we present the plot of the polymer weight loss against the *R*_a_ between each of the solvents and the MWCNTs. We can observe that there are two different trends present in the plot: one encompassing DCM, toluene, and acetone (circled in red), and a second trend with *o*-DCB, NMP and DMF (circled in blue). The observation of two separate trends is consistent with a previous report by Lim *et al.*^14^ who applied the HSP theory to predict the dispersability of MWCNTs in several organic solvents. The recurring observation of two separate tendencies instead of a single trend can be explained due to the limitations of the HSP theory to model electron exchange interactions accurately.^24^ The electron charge transfer from *o*-DCB, NMP, and DMF to graphitic materials, such as graphene, has been reported previously.^25^ For this reason, it is not surprising that this model is overestimating the energetic interactions between these solvents and MWCNTs.

If we compare the trend obtained from the Hildebrand SEBSP difference (Fig. 2a) with the two trends observed for the Hansen SEBSP distance (Fig. 2b), we can note that the latter model can provide further insights to the nature of the energetic interactions between the solvents and MWCNTs. For example, the trend observed in Fig. 2b for the samples prepared in *o*-DCB, NMP, and DMF (circled in blue) is more consistent with the experimental results than predictions based on the Hildebrand SEBSP difference. A similar behaviour is observed for toluene, DCM and acetone (circled in red). We attribute this improvement to the separation of the surface energy into different intermolecular forces providing a more accurate representation of the interaction between the solvents and MWCNTs.

In summary, we have introduced the application of the SEBSP theories to estimate the degree of polymer functionalization of MWCNTs in different solvents. In particular, we found that by complementing the Hildebrand SEBSP with the Hansen SEBSP theory we could obtain a good prediction of the degree of polymer functionalization of MWCNTs in the different solvents used for this study. The large database of Hansen solubility parameters for organic solvents available in the literature and the simplicity of these theories makes them a powerful qualitative guideline for future development of functionalization procedures. We believe that this breakthrough will enable the rational design of functionalization procedures for a wide range of MWCNT-based nanomaterials.

## Conflicts of interest

There are no conflicts to declare.

## Supplementary Material

Supplementary information

## References

[cit1] Tasis D., Tagmatarchis N., Bianco A., Prato M., Tasis D., Tagmatarchis N., Bianco A., Prato M. (2006). Chem. Rev..

[cit2] Zhao Y.-L., Stoddart J. F. (2009). Acc. Chem. Res..

[cit3] O’Connell M. J., Boul P., Ericson L. M., Huffman C., Wang Y., Haroz E., Kuper C., Tour J., Ausman K. D., Smalley R. E. (2001). Chem. Phys. Lett..

[cit4] Meuer S., Braun L., Schilling T., Zentel R. (2009). Polymer.

[cit5] Shin H., Min B. G., Jeong W., Park C. (2005). Macromol. Rapid Commun..

[cit6] Oliveira E. Y. S., Bode R., Escárcega-Bobadilla M. V., Zelada-Guillén G. A., Maier G. (2016). New J. Chem..

[cit7] Lee S. H., Park J. S., Lim B. K., Kim S. O. (2008). J. Appl. Polym. Sci..

[cit8] Tamesue S., Numata M., Kaneko K., James T. D., Shinkai S. (2008). Chem. Commun..

[cit9] O’Connell M. J., Boul P., Ericson L. M., Huffman C., Wang Y., Haroz E., Kuper C., Tour J., Ausman K. D., Smalley R. E. (2001). Chem. Phys. Lett..

[cit10] Schröder J. (1998). Eur. Coat. J..

[cit11] Bergin S. D., Sun Z., Rickard D., Streich P. V., Hamilton J. P., Coleman J. N. (2009). ACS Nano.

[cit12] Detriche S., Nagy J. B., Mekhalif Z., Delhalle J. (2009). J. Nanosci. Nanotechnol..

[cit13] Detriche S., Zorzini G., Colomer J.-F., Fonseca A., Nagy J. B. (2008). J. Nanosci. Nanotechnol..

[cit14] Lim H. J., Lee K., Cho Y. S., Kim Y. S., Kim T., Park C. R. (2014). Phys. Chem. Chem. Phys..

[cit15] Dutta M., Nicolosi V., Obratzsova E., Koós A. A., Crossley A., Grobert N. (2011). Mater. Express.

[cit16] Hughes J. M., Aherne D., Coleman J. N. (2013). J. Appl. Polym. Sci..

[cit17] Ma J., Larsen R. M. (2013). ACS Appl. Mater. Interfaces.

[cit18] May P., Khan U., Hughes J. M., Coleman J. N. (2012). J. Phys. Chem. C.

[cit19] Bahr J. L., Mickelson E. T., Bronikowski M. J., Smalley R. E., Tour J. M. (2001). Chem. Commun..

[cit20] Ausman K. D., Piner R., Lourie O., Ruoff R. S., Korobov M. (2000). J. Phys. Chem. B.

[cit21] Imre A., Van Hook W. A. (1996). J. Phys. Chem. Ref. Data.

[cit22] Dawkins J. V. (1976). J. Polym. Sci., Polym. Phys. Ed..

[cit23] Hansen C. M., Hansen solubility parameters: a user's handbook, CRC Press, Boca Raton FL, 2nd edn, 2007.

[cit24] Louwerse M. J., Maldonado A., Rousseau S., Moreau-Masselon C., Roux B., Rothenberg G. (2017). ChemPhysChem.

[cit25] Liu W. W., Wang J. N., Wang X. X. (2012). Nanoscale.

