# Supplementary material for: The application of the surface energy based solubility parameter theory for the rational design of polymer-functionalized MWCNTs
Source: Phys Chem Chem Phys. 2019 Feb 14;21(10):5331–4. doi: 10.1039/c8cp07411a (PMC6530085; doi:10.1039/c8cp07411a)
Supplement: Supplementary file 1 [file CP-021-C8CP07411A-s001.pdf]

Electronic Supplementary Material (ESI) for Physical Chemistry Chemical Physics.
This journal is © the Owner Societies 2019

The application of the Surface Energy Based Solubility Parameter Theory for the rational design of polymer-functionalized CNTs

Pablo Quijano Velasco[a], Kyriakos Porfyrakis[a] and Nicole Grobert[a,b]

[a]Department of Materials, University of Oxford, Parks Road, Oxford, OX1 3PH, UK.

[b]Williams Advanced Engineering, Grove, Oxfordshire, OX12 0DQ, UK

E-mail: pablo.quijanovelasco@materials.ox.ac.uk

nicole.grobert@materials.ox.ac.uk; Tel +44 (0) 1865 283720

kyriakos.porfyrakis@materials.ox.ac.uk; Tel: +44 (0)1865 273724

Supporting information

EXPERIMENTAL

Materials and characterization details

Tetrahydrofuran (HPLC grade ≥99.9%), Styrene (containing 4-Tertbutyl catechol >99%), 2,2-bipyridine (99%), 2-(4-bromomethyl)phenylpropionic acid (96%) and copper (I) bromide (99.999% trace metal basis) used for the synthesis of polystyrene by atom transfer polymerization procedure were purchased from Sigma-Aldrich.

The solvents used for the functionalization experiments were purchased from the following providers. Dichloromethane (DCM,≥99.98%), dimethylformamide (DMF, HPLC grade ≥99.99%) and toluene (HPLC grade ≥99.99%) were purchased from Fischer Scientific. 1-Methyl-2-pyrrolidone (HPLC grade ≥99%) and acetone (Analytical Grade ≥99%) were purchased from Sigma-Aldrich. Orto-Dichlorobenzene (o-DCB,Reagent grade ≥99%) was purchased from Honeywell. The multi-walled carbon nanotubes used for this study were purchased from Nanocyl (Nanocyl7000) and used as received. DCTB (Trans-2-[3-(4-tert-Butylphenyl)-2-methyl-2-propenylidene]malononitrile, 98%) and Silver trifluoroacetate (98%) used for MALDI-TOF characterization were purchased from Sigma Aldrich and used as received.

Solution nuclear magnetic resonance (NMR) spectra were recorded at 298K on a 400 MHz Bruker AVANCE III HD. The samples were prepared using deuterated chloroform (99.8% D) purchased from Sigma-Aldrich.

The MALDI-TOF spectrum for the molecular weight characterization was recorded on a Bruker Microflex LT spectrometer. The samples were prepared using a polymer solution with a concentration of 2 mg/mL in THF, a 0.6 M solution of DCTB in THF and 0.06 M solution of silver trifluoracetate in THF. The silver trifluoroacetate to promote the ionization of the polymer[1]. For the analysis 10 µL of each solution were mixed in a vial. Afterwards 0.5 µL of the mixture was drop casted in the MALDI-TOF target.

Themogravimetric analysis was performed under nitrogen atmosphere in a Perkin Elmer Diamond TG/DTA6300 using a heating rate of 10 °C/minute.

Gel permeation chromatography (GPC) data was obtained using a Shimadzu LC-20AD instrument with HPLC grade THF as the eluent flowing at 1.0 mL/min at 30 °C and calibrated using near monodisperse polystyrene standards.

Transmission Electron Microscopy images were recorded on a JEOL 3000F TEM microscope operating in an accelerating voltage of 300 kV. The samples were prepared by drop casting MWCNTs dispersions in acetone into lacey-carbon copper grids.

Hansen solubility parameters and calculation of the surface energy based solubility parameters

The calculation of the HSP surface energy based solubility parameters was performed according to Bergin et al. methodology[2]. In summary, to obtain the values of the SEBSP of the solvents Bergin et al. proposed the following equation:

$$E_{S,i} = \frac{\propto_i \delta_i^2}{\propto_D \delta_D^2 + \propto_P \delta_P^2 + \propto_H \delta_H^2} E_{S,Total} \qquad (6)$$

Where $E_{S,Total}$ is the surface energy of the substance, δ_i are the Hansen solubility parameters (HSP) of the substance, and α_i are empirical constants that are equal to $\alpha_D = 1$ and $\alpha_{P,H} = 0.632$. In addition, the total surface energy ($E_{S,Total}$) of the solvents were calculated using the following equation:

$$E_{S,Total} = \gamma + T\Delta S_s \qquad (7)$$

Where γ is the surface tension of the solvent and ΔS_s is the solvents surface entropy that is equal to 0.1 mJ m^{-2} K^{-1} and can be considered constant for any organic solvent[2,3]. Finally, the surface energy based solubility parameter was calculated as follows:

$$\partial_{i=D,P,H} = \sqrt{E_{S,i}}$$

In the following table a summary of the HSP and surface energy based solubility parameters (SEBSP) of the solvents and MWCNTs is presented.

Substance	δ_D ($MPa^{1/2}$)	δ_P ($MPa^{1/2}$)	δ_H ($MPa^{1/2}$)	γ (mJ/m^2)	$E_{S,Total}$ (mJ/m^2)	∂_D ($mJ^{1/2}/m$)	∂_P ($mJ^{1/2}/m$)	∂_H ($mJ^{1/2}/m$)
o-DCB	19.2[a]	6.3 [a]	3.3 [a]	35.55[c]	67.39	7.9	2.0	1.1
NMP	18 [a]	12.3 [a]	7.2 [a]	42.51 [c]	71.81	7.1	3.9	2.3
DMF	17.4 [a]	13.7 [a]	11.3 [a]	34.43 [c]	63.73	6.2	3.9	3.2
DCM	18.2 [a]	6.3 [a]	6.1 [a]	27.84 [c]	57.14	7.1	1.9	1.9
Toluene	18 [a]	1.4 [a]	2 [a]	27.93[c]	57.23	7.5	0.5	0.7
Acetone	15.5 [a]	10.4 [a]	7 [a]	23.04 [c]	52.34	6.1	3.2	2.2
MWCNTs	21.3[b]	6.8 [b]	3.2 [b]	-	114[d]	10.3	2.6	1.2

Table S1. HSP, surface tension, surface energy and SEBSP of the solvents used during this study. [a] The values of the HSP of the solvent were taken form from the textbook *Hansen Solubility Parameters: A User's Handbook*[4]. [b] The HSP of the MWCNTs were taken from Lim et al.[5] study. [c] The surface tension values were taken from the textbook *Thermophysical Properties of Chemicals and Hydrocarbons*[6] [d] The surface energy of the MWCNTs were taken from Menzel et al.[7] report.

Synthesis of polystyrene by ATRP

The methodology for the synthesis of polystyrene was based on a procedure reported previously[8]. In a typical experiment 2-(4-bromomethyl)phenylpropionic acid (1.05 g, 4.34 mmol), copper (I) bromide (0.62g, 4.34 mmol), and 2,2-bipyridine (1.35g, 8.68 mmol) were added to a three-necked round bottom flask. The flask was fitted with a septum and the solid powders were flushed in vacuum and purged with argon in a Schlenck line, the evacuation procedure was repeated three times. Afterwards, 3.5 mL of DMF were added and the mixture was flushed in vacuum and purged with argon in a Schlenck line, the evacuation procedure was repeated three times. Under a nitrogen (N_2) flow, 20 mL of deoxyganated styrene were added with a syringe to the reaction vessel and it was placed in an oil bath at 110 C for 3 hours. Afterwards, the reaction mixture was stopped and diluted in 200 mL of THF. The mixture was vacuum filtered through a short neutral alumina column to remove the solid catalyst particles and the mixture was concentrated under vacuum and precipitated in methanol twice. The polymer was separated by vacuum filtration obtaining a light blue powder. ^{1}H NMR (Fig. S2) was consistent with the expected polystyrene spectrum and MALDI-TOF mass spectrometry (Fig. S3) showed that the difference of the molecular masses is equal to the expected mass of styrene monomers. ^{1}H NMR (450MHz, $CDCl_3$): 7.10-6.50 ppm (broad, aromatic H), 2.5-1.1 ppm (broad, $CH+CH_2$). The polymer number average molecular weight (Mn= 3333 g/mol) and weight average molecular weight (Mw=3609) were determined by GPC. MALDI-TOF mass spectrometry of the polymer showed a molecular weight separation of 104 g/mol consistent with the molecular weight of the styrene molecules (Figure S2).

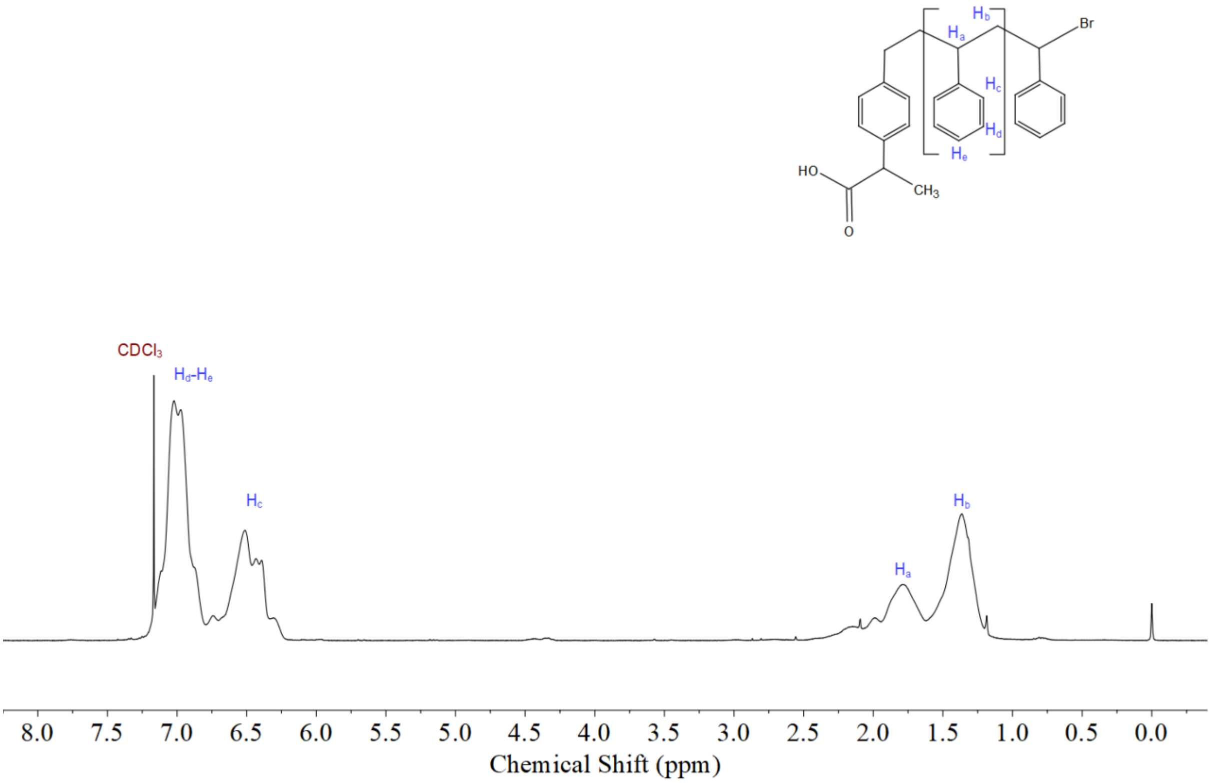

Figure S1 NMR spectrum of the polystyrene used during this study. The 1H signals corresponding to the aliphatic and aromatic hydrogens of the styrene monomers are tagged in blue.

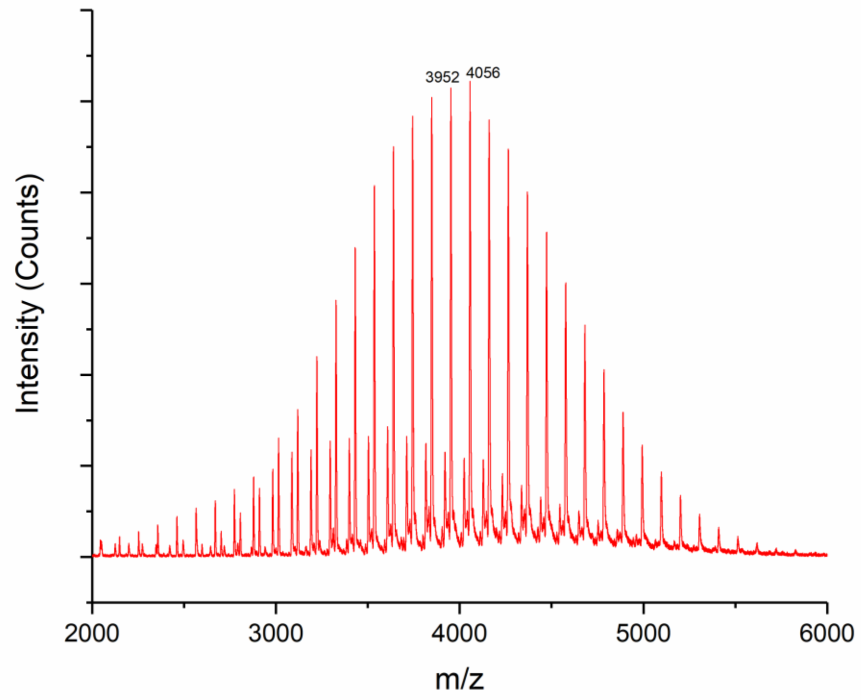

Figure S2 MALDI-TOF mass spectrum of the polystyrene used during this study. The two most intense mass counts are highlighted.

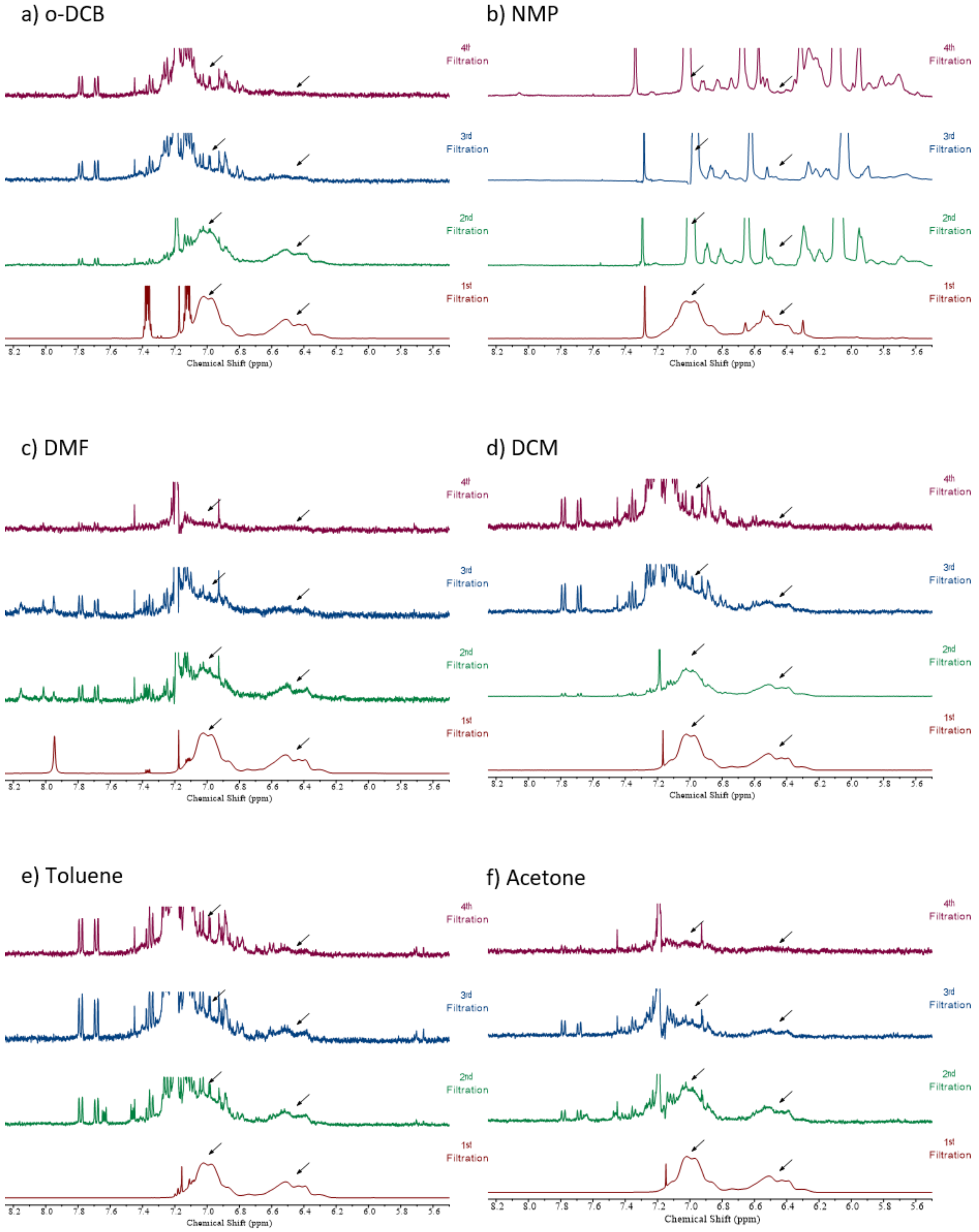

Figure S3 NMR of the filtrate after each washing in the functionalization procedure for the different solvent media used in this study. The arrows point to the position of polystyrene's aromatic protons. No polymer can be observed after the fourth redispersion. Solvents used: a) orto-Dichlorobenzene b) N-methyl pyrrolidone c) Dimethylformamide d) Dichloromethane e) Toluene, and f) Acetone.

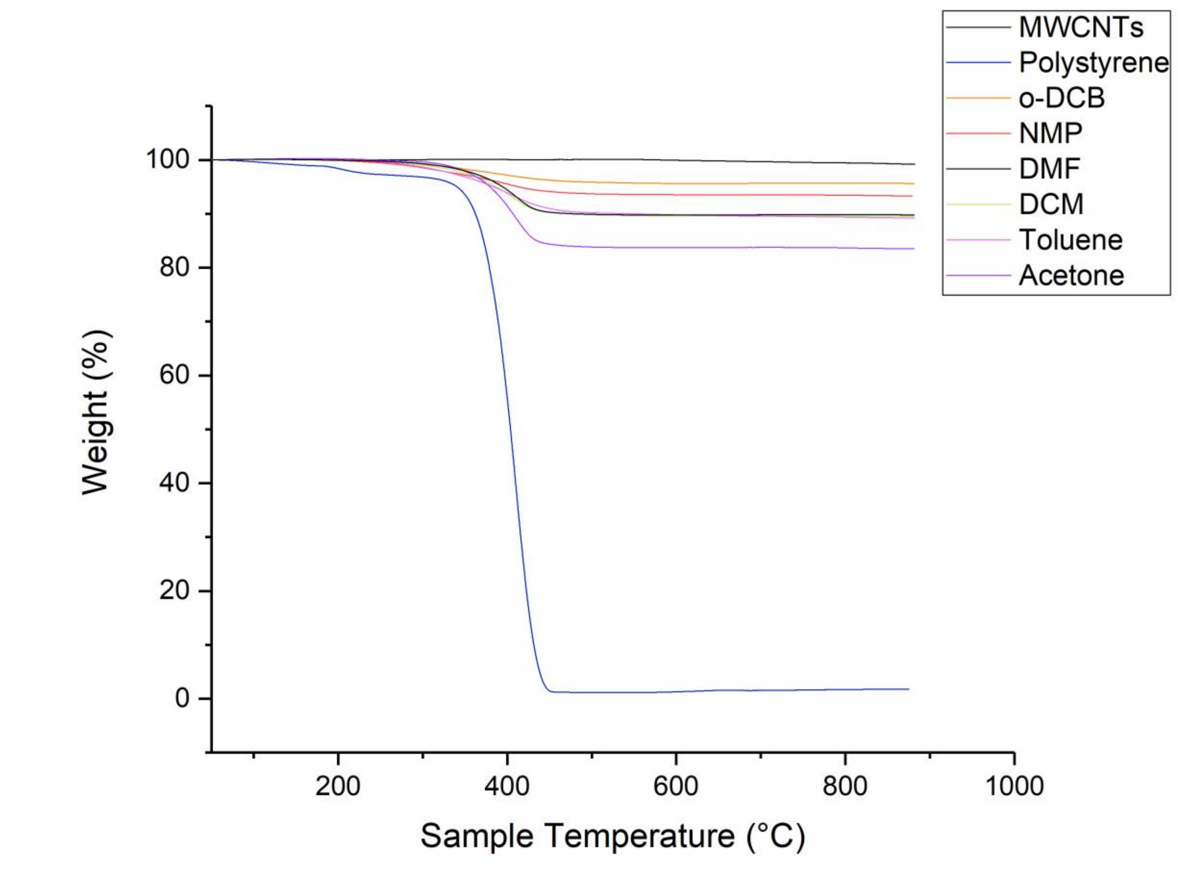

Figure S4 Representative TGA curves of MWCNTs, polystyrene and polymer functionalized MWCNTs. Pristine MWCNTs do not present a significant weight loss when heated to 900 C, while polystyrene fully decompose after 650 °C. Polymer functionalized MWCNTs show a characteristic weight loss depending on the solvent used during the functionalization procedure ranging from approximately 5% to 16% of the weight of the sample.

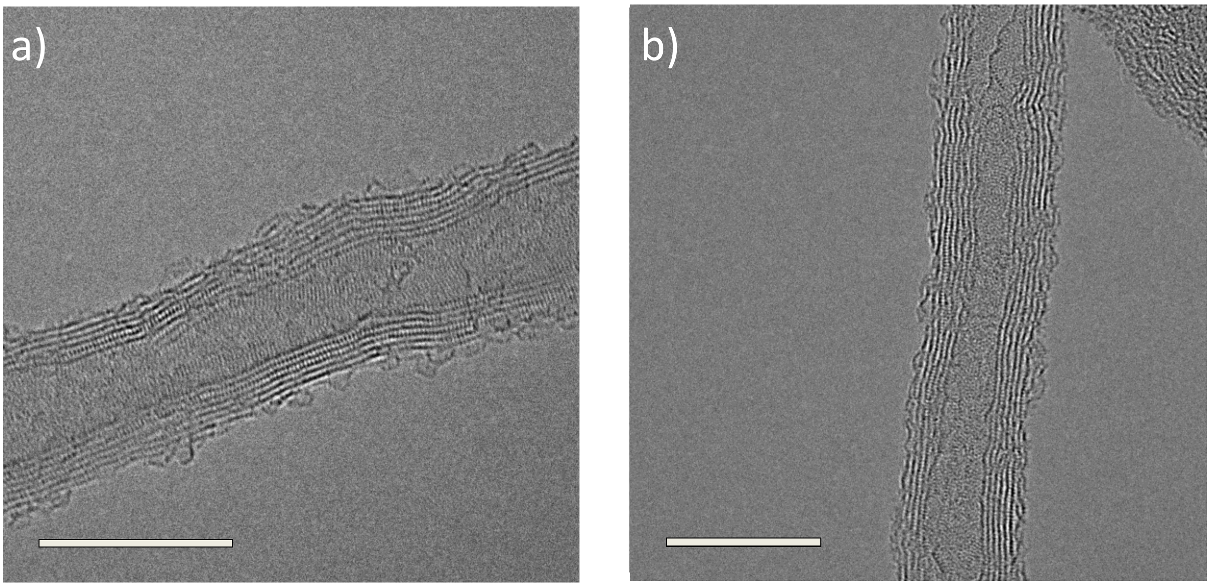

Figure S5 TEM image of a) Pristine MWCNTs and b) MWCNTs functionalized in acetone. In both samples, the MWCNTs present non-crystalline structures adsorbed to the walls of the CNTs.

References

1 A. Tintaru, C. Chendo, T. N. T. Phan, M. Rollet, L. Giordano, S. Viel, D. Gigmes and L. Charles, , DOI:10.1021/ac400375d.

2 S. D. Bergin, Z. Sun, D. Rickard, P. V Streich, J. P. Hamilton and J. N. Coleman, *ACS Nano*, 2009, **3**, 2340–2350.

3 J. Lyklema, *Colloids Surfaces A Physicochem. Eng. Asp.*, 1999, **156**, 413–421.

4 C. M. Hansen, *Hansen solubility parameters : a user's handbook*, CRC Press, Boca Raton FL, Second Edi., 2007.

5 H. J. Lim, K. Lee, Y. S. Cho, Y. S. Kim, T. Kim and C. R. Park, *Phys. Chem. Chem. Phys.*, 2014, **16**, 17466.

6 C. L. Yaws, *Thermophysical Properties of Chemicals and Hydrocarbons*, Elsevier, Second Edi., 2014.

7 R. Menzel, A. Lee, A. Bismarck and M. S. P. Shaffer, *Langmuir*, 2009, **25**, 8340–8348.

8 P. Y. Keng, I. Shim, B. D. Korth, J. F. Douglas and J. Pyun, *ACS Nano*, 2007, **1**, 279–292.
